# SARS-CoV-2 infection, disease and transmission in domestic cats

**DOI:** 10.1080/22221751.2020.1833687

**Published:** 2020-10-25

**Authors:** Natasha N. Gaudreault, Jessie D. Trujillo, Mariano Carossino, David A. Meekins, Igor Morozov, Daniel W. Madden, Sabarish V. Indran, Dashzeveg Bold, Velmurugan Balaraman, Taeyong Kwon, Bianca Libanori Artiaga, Konner Cool, Adolfo García-Sastre, Wenjun Ma, William C. Wilson, Jamie Henningson, Udeni B. R. Balasuriya, Juergen A. Richt

**Affiliations:** a Department of Diagnostic Medicine/Pathobiology, College of Veterinary Medicine, Kansas State University, Manhattan, KS, USA; b Louisiana Animal Disease Diagnostic Laboratory and Department of Pathobiological Sciences, School of Veterinary Medicine, Louisiana State University, Baton Rouge, LA, USA; c Department of Microbiology, Icahn School of Medicine at Mount Sinai, New York, NY, USA; d Global Health and Emerging Pathogens Institute, Icahn School of Medicine at Mount Sinai, New York, NY, USA; e Department of Medicine, Division of Infectious Diseases, Icahn School of Medicine at Mount Sinai, New York, NY, USA; f The Tisch Cancer Institute, Icahn School of Medicine at Mount Sinai, New York, NY, USA; g Arthropod Borne Animal Disease Research Unit, Agricultural Research Service, United States Department of Agriculture, Manhattan, KS, USA

**Keywords:** SARS-CoV-2, COVID-19, felines, cats, susceptibility, transmission

## Abstract

Severe Acute Respiratory Syndrome Coronavirus 2 (SARS-CoV-2) is the cause of Coronavirus Disease 2019 (COVID-19) and responsible for the current pandemic. Recent SARS-CoV-2 susceptibility studies in cats show that the virus can replicate in these companion animals and transmit to other cats. Here, we present an in-depth study of SARS-CoV-2 infection, disease and transmission in domestic cats. Cats were challenged with SARS-CoV-2 via intranasal and oral routes. One day post challenge (DPC), two sentinel cats were introduced. Animals were monitored for clinical signs, clinicopathological abnormalities and viral shedding. *Postmortem* examinations were performed at 4, 7 and 21 DPC. Viral RNA was not detected in blood but transiently in nasal, oropharyngeal and rectal swabs and bronchoalveolar lavage fluid as well as various tissues. Tracheobronchoadenitis of submucosal glands with the presence of viral RNA and antigen was observed in airways of the infected cats. Serology showed that both, principals and sentinels, developed antibodies to SARS-CoV-2. All animals were clinically asymptomatic during the course of the study and capable of transmitting SARS-CoV-2 to sentinels. The results of this study are critical for understanding the clinical course of SARS-CoV-2 in a naturally susceptible host species, and for risk assessment.

## Introduction

Coronaviruses are enveloped single-stranded, positive-sense RNA viruses that belong to the order *Nidovirales* in the family *Coronaviridae*, subfamily *Orthocoronavirinae*, and are comprised of four genera: *Alphacoronavirus, Betacoronavirus, Gammacoronavirus, and Deltacoronavirus* [1]. The Severe Acute Respiratory Syndrome-related coronaviruses (SARS-CoV and SARS-CoV-2), and the Middle East Respiratory Syndrome coronavirus (MERS-CoV) belong to the genus *Betacoronavirus* [2,3]. Alpha- and betacoronaviruses infect mammals and cause important respiratory, enteric, and systemic infectious diseases of humans, cattle, pigs, cats, dogs, horses, and camels [1,4,5]. Importantly, coronaviruses can occasionally cross the species barriers [6,7].

Bats have been identified as a reservoir species for zoonotic coronaviruses including those causing important human epidemics, namely SARS-CoV in 2002–2003 and MERS-CoV since 2012 [6]. Camels have since been shown to serve as the primary intermediate and reservoir host for MERS-CoV, causing continued zoonotic animal-to-human transmissions [8]. During the SARS-CoV epidemic, infected domestic cats were identified from households of SARS-CoV positive patients, and both cats and ferrets were subsequently experimentally shown to be easily infected and to transmit SARS-CoV [9,10].

SARS-CoV-2 is the cause of Coronavirus Disease 2019 (COVID-19) and responsible for the current global pandemic [11]. A zoonotic transmission event amplified at a seafood and animal market in Wuhan, Hubei Province, China, is suspected to be the site of the first significant outbreak in humans [12], with bats and/or pangolins being speculated as the potential origin species based on the sequence homology of coronaviruses isolated from these animals [11,13,14].

Since the outbreak of SARS-CoV-2 was first identified in December of 2019, it has been demonstrated that SARS-CoV-2 can naturally and experimentally infect several animal species [15–17]. There have been multiple case reports of natural transmission of the virus from COVID-19 patients to dogs and cats, infection of “big cats” (i.e. a lion and tigers) at the Bronx Zoo, and infection of mink on farms in The Netherlands, Denmark, Spain, and the United States [17–19]. In a recent animal susceptibility study, dogs, cats, ferrets, pigs, chickens and ducks were experimentally infected with SARS-CoV-2 [20]. The results from that study show that both cats and ferrets were efficiently infected and could transmit the virus, dogs showed low susceptibility, while pigs and avian species were non permissive hosts. In addition, non-human primates (NHPs), hamsters and hACE2 transgenic or adenovirus transduced mice have also been evaluated as potential animal models for SARS-CoV-2 and seem to be highly susceptible showing mild to severe clinical signs [15,21].

The close association between humans and animals including companion animals, livestock and wildlife species, raises concerns regarding the potential risks of transmission of SARS-CoV-2 from COVID-19 patients to animals (“reverse zoonosis”), and the potential role infected animals could play in perpetuating the spread of the disease [16,19]. Therefore, further research of SARS-CoV-2 infection in various animal species is needed in order to identify susceptible hosts and to better understand the infection, disease, clinical course and transmission capabilities of susceptible animal species. This knowledge is important for risk assessment, implementing mitigation strategies, addressing animal welfare issues, and to develop preclinical animal models for evaluating drug and vaccine candidates for COVID-19.

Here, we present an in-depth study of SARS-CoV-2 infection, associated disease and transmission in domestic cats. Clinical evaluation of weight, body temperature, blood parameters, serology, viral RNA shedding and RNA distribution in tissues and organ systems, and associated pathological findings are presented and discussed.

## Material and methods

### Cells and virus

Vero E6 cells (ATCC^®^ CRL-1586^™^, American Type Culture Collection, Manassas, VA, USA) were used for virus propagation and titration. Cells were cultured in Dulbecco’s Modified Eagle’s Medium (DMEM, Corning, New York, N.Y, USA), supplemented with 5% fetal bovine serum (FBS, R&D Systems, Minneapolis, MN, USA) and antibiotics/antimycotics (ThermoFisher Scientific, Waltham, MA, USA), and maintained at 37°C under a 5% CO_2_ atmosphere. The SARS-CoV-2 USA-WA1/2020 strain was acquired from BEI Resources (Manassas, VA, USA) and passaged three times in Vero E6 cells to establish a stock virus (1 × 10^6^ TCID_50_/ml) for inoculation of animals. This stock virus was sequenced by next generation sequencing (NGS) using the Illumina MiSeq and its consensus sequence was found to be 100% homologous to the original USA-WA1/2020 strain (GenBank accession: MN985325.1). To determine infectious virus titre, 10-fold serial dilutions were performed on Vero E6 cells. The presence of cytopathic effects (CPE) after 96 h incubation was used to calculate the 50% tissue culture infective dose (TCID_50_)/ml using the Spearman-Karber method [22].

### Animals and experimental design

#### Ethics statement for use of animals

All animal studies and experiments were approved and performed under the Kansas State University (KSU) Institutional Biosafety Committee (IBC, Protocol #1460) and the Institutional Animal Care and Use Committee (IACUC, Protocol #4390) in compliance with the Animal Welfare Act. All animal and laboratory work were performed in biosafety level-3+ and −3Ag laboratories and facilities in the Biosecurity Research Institute at KSU in Manhattan, KS, USA.

#### Virus challenge of animals

**Figure 1.**
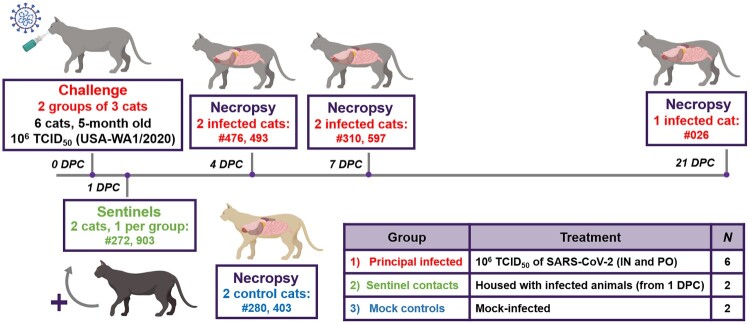
Study design. Ten cats were placed into three groups. Group 1 (principal infected animals) consisted of six cats (three cats/housing unit) and was inoculated via intranasal (IN) and oral (PO) routes simultaneously with a total dose of 1 × 10^6^ TCID_50_ of SARS-CoV-2 in 2 ml DMEM. The cats in Group 2 (*n *= 2; sentinel contact animals) and Group 3 (*n *= 2; mock control animals) were housed in a separate room. At 1-day post challenge (DPC), the two cats in Group 2 were co-mingled with the principal infected animals in Group 1 (one cat per cage) and served as sentinel contact controls. The remaining two cats in Group 3 were housed in a separate room and served as mock-infected negative controls. Principal infected animals were euthanized and necropsied at 4 (*n *= 2), 7 (*n *= 2) and 21 (*n *= 1) DPC to evaluate the course of disease. The two negative control animals in Group 3 were euthanized and necropsied at 3 DPC. The remaining three animals from Group 1 (one principal infected animal) and Group 2 (two sentinel contact animals) were maintained for future re-infection studies.

Ten 4.5- to 5-month-old intact male cats were acclimated for seven days to BSL-3Ag biocontainment prior to experimental procedures with feed and water *ad libitum*. These were antibody profile defined/specific pathogen free (APD/SPF) animals with no detectable antibody titres to feline herpesvirus (rhinotracheitis), feline calicivirus, feline panleukopenia virus, feline coronaviruses, feline immunodeficiency virus, *Chlamydia felis* and *Toxoplasma gondii* obtained from Marshall BioResources (North Rose, New York, USA). The cats were placed into three groups (Figure 1). Group 1 (principal infected animals) consisted of six cats (three cats per housing unit), and was inoculated simultaneously via the intranasal and oral routes with a total dose of 1 × 10^6^ TCID_50_ of SARS-CoV-2 in a total volume of 2 ml DMEM medium (0.5 ml per nostril and 1 ml oral). The cats in Group 2 (*n* = 2; sentinel contact animals) and Group 3 (*n* = 2; mock control animals) were housed in a separate room (Figure 1). Mock-infected cats (Group 3) were administered 2 ml DMEM via the intranasal and oral routes similar to Group 1 animals. At 1-day post challenge (DPC), the two cats in Group 2 were co-mingled with the principal infected animals in Group 1 (one cat per housing unit), and served as sentinel contact controls. The remaining two cats in Group 3 remained housed in a separate room and served as mock-infected negative controls. Principal infected animals were euthanized for *postmortem* examinations at 4 (*n* = 2), 7 (*n* = 2) and 21 (*n* = 1) DPC to evaluate the course of disease. The two negative control animals in Group 3 were euthanized for *postmortem* examinations at 3 DPC. The remaining three animals from Group 1 (one principal infected animal) and Group 2 (two sentinel contact animals) were maintained for future re-infection studies, and not terminated as part of this study.

### Clinical evaluations and sample collection

Cats were observed daily for clinical signs, such as: fever, anorexia, lethargy, respiratory distress, inappetence, depression, recumbency, coughing, sneezing, diarrhea/loose stool, vomiting and others. Weights of all cats were recorded on bleed days. Blood and serum were collected from all cats, including sentinel contact controls, on −1 DPC prior to infection, and on days 1, 3, 5, 7, 10, 14 and 21 DPC via venipuncture of the cephalic vein under anesthesia or during terminal bleeding by cardiac puncture. Nasal, oropharyngeal and rectal swabs were also collected on −1, 1, 3, 5, 7, 10, 14 and 21 DPC in 2 ml of viral transport medium (VTM; DMEM; Corning,) with antibiotics/antimycotic (ThermoFisher). Swabs were vortexed and supernatant aliquoted directly into cryovials or into RLT buffer (Qiagen, Germantown, MD, USA) and stored at −80°C until further analysis.

A full *postmortem* examination was performed for each cat at the indicated time-points and gross changes (if any) were recorded. Tissues were collected either in 10% neutral-buffered formalin (Fisher Scientific, Waltham, MA, USA), or as fresh tissues which were then frozen at −80°C. A *postmortem* examination protocol was developed to collect the upper and lower respiratory tract, central nervous system (brain and cerebral spinal fluid [CSF]; Figure S1A), gastrointestinal (GI) tract as well as accessory organs. The lungs were removed *in toto* including the trachea, and the main bronchi were collected at the level of the bifurcation and at the entry point into the lung lobe (Figure S1B). Lung lobes were evaluated based on gross pathology and collected and sampled separately (Figure S1C). Bronchoalveolar lavage fluid (BALF), nasal wash and urine were also collected during *postmortem* examination and stored at −80°C until analysed. Fresh frozen tissue homogenates were prepared by thawing tissue and placing 200 mg (± 50 mg) of minced tissue in a tube containing 1 ml DMEM culture medium and a steel bead (Qiagen). Homogenization was performed with the TissueLyser LT (Qiagen) for 30 s at 30 hertz and repeated 3 times. Supernatant was retained after centrifugation for RNA extraction and reverse transcription quantitative PCR (RT-qPCR).

### Blood cell counts

Complete blood cell counts were performed using fresh EDTA blood samples run on an automated VetScan HM5 Hematology Analyser (Abaxis Inc., Union City, CA) according to the manufacturer’s recommended protocol using the VetScan HM5 reagent pack and recommended calibration controls. Blood cell analysis included: complete white blood cells, lymphocytes, monocytes, neutrophils, eosinophils, basophils, red blood cells, hematocrit, hemoglobin and platelets.

### Serum biochemistry

Serum chemistry was performed using an automated VetScan VS2 Chemistry Analyser (Abaxis) according to the manufacturer’s recommended protocol. The Comprehensive Diagnostic Profile reagent rotor was used to perform complete chemistry and electrolyte analysis on 14 blood components: alkaline phosphatase, creatine, globulin, phosphorous, glucose, blood urea nitrogen, sodium, potassium, calcium, alanine aminotransferase, amylase, albumin, total bilirubin, total protein. Briefly, 100 µl of serum was added to the sample port of the reagent rotor, which was subsequently run in the machine.

### RNA extraction and reverse transcription quantitative PCR (RT-qPCR)

SARS-CoV-2-specific RNA was detected using a RT-qPCR assay. Briefly, tissue homogenates in DMEM, blood, CSF, BALF, urine, and nasal, oropharyngeal and rectal swabs in VTM were mixed with an equal volume of RLT RNA stabilization/lysis buffer (Qiagen, Germantown, MD, USA), and 200μl of sample lysate was then used for extraction using a magnetic bead-based nucleic acid extraction kit (GeneReach USA, Lexington, MA) on an automated Taco^TM^ mini nucleic acid extraction system (GeneReach) according to the manufacturer’s protocol with the following modifications: beads were added to the lysis buffer in the first well followed by the RLT sample lysate, then by the addition of 200 μl molecular grade isopropanol (ThermoFisher), and finally, the last wash buffer B was replaced with 200 proof molecular grade ethanol (ThermoFisher). Extraction positive controls (IDT, IA, USA; 2019-nCoV_N_Positive Control, diluted 1:100 in RLT) and negative controls were employed.

Quantification of SARS-CoV-2 RNA was performed using the N2 SARS-CoV-2 primer and probe sets (see: https://www.idtdna.com/pages/landing/coronavirus-research-reagents/cdc-assays) in a RT-qPCR protocol established by the CDC for the detection of SARS-CoV-2 nucleoprotein (N)-specific RNA (https://www.fda.gov/media/134922/download). This protocol has been validated in our lab for research use, using the qScript XLT One-Step RT-qPCR Tough Mix (Quanta BioSsciences, Beverly, MA, USA) on the CFX96 Real-Time thermocycler (BioRad, Hercules, CA, USA) using a 20-minute reverse transcription step and 45 cycle PCR in a 20 μl reaction volume. A reference standard curve method using a 10-point standard curve of quantitated viral RNA (USA-WA1/2020 isolate) was used to quantify RNA copy number. RT-qPCR was performed in duplicate wells with a quantitated PCR positive control (IDT, IA, USA; 2019-nCoV_N_Positive Control, diluted 1:100) and four non-template control (NTC) on every plate. A positive Ct cut-off of 40 cycles was used. Data are presented as the mean and standard deviation of the calculated N gene copy number per ml of liquid sample or per mg of a 20% tissue homogenate.

### Virus neutralizing antibodies

Virus neutralizing antibodies in sera were determined using microneutralization assay. Briefly, serum samples were initially diluted 1:10 and heat-inactivated at 56°C for 30 min while shaking. Subsequently, 100 μl per well of serum samples in duplicates were subjected to 2-fold serial dilutions starting at 1:20 through 1:2560 in 100 μl culture media. Then, 100 μl of 100 TCID_50_ of SARS-CoV-2 virus in DMEM culture media was added to 100 μl of the sera dilutions and incubated for 1 h at 37°C. The 200 μl per well of virus serum mixture was then cultured on Vero E6 cells in 96-well plates. The corresponding SARS-CoV-2-negative cat serum, virus only and media only controls were also included in the assay. The neutralizing antibody titre was recorded as the highest serum dilution at which at least 50% of wells showed virus neutralization (NT_50_) based on the appearance of CPE observed under a microscope at 72 h post infection.

### Detection of SARS-CoV-2 antibodies by indirect ELISA

To detect SARS-CoV-2 antibodies in sera, indirect ELISAs were performed with the recombinant viral proteins, nucleocapsid (N) and the receptor-binding domain (RBD), which were produced in-house. The N protein was produced in *E. coli* with a C-terminal His-Tag, and the RBD was expressed in mammalian cells with a C-terminal Strep-Tag; they were purified using either Ni-NTA (ThermoFisher) or Strep-Tactin (IBA Lifesciences, Goettingen, Germany) columns, respectively, according to the manufacturers’ instructions.

For indirect ELISAs, wells were coated with 100 ng of the respective protein in 100 μl per well coating buffer (Carbonate–bicarbonate buffer, catalogue number C3041, Sigma-Aldrich, St. Louis, MO, USA), then covered and incubated overnight at 4°C. The next day, the plates were washed two times with phosphate buffered saline (PBS [pH=7.2–7.6]; catalogue number P4417, Sigma-Aldrich), blocked with 200 μl per well casein blocking buffer (Sigma-Aldrich, catalogue number B6429) and incubated for 1 h at RT. The plates were then washed three times with PBS-Tween-20 (PBS-T; 0.5% Tween-20 in PBS). Serum samples were pre-diluted 1:400 in casein blocking buffer, then 100 μl per well was added to the ELISA plate and incubated for 1 h at RT. The wells were washed three times with PBS-T, then 100 μl of HRP-labelled goat anti-feline IgG (H + L) secondary antibody (ThermoFisher, catalogue number A18757) diluted 1:2500 was added to each well and incubated for 1 h at RT. After 1 h, plates were washed five times with PBS-T, and 100 μl of TMB ELISA Substrate Solution (Abcam, catalogue number ab171525, Cambridge, MA, USA) was added to all wells of the plate. Following incubation at RT for 5 min, the reaction was stopped by adding 100 μl Stop Solution for TMB Substrate (Abcam, catalogue number ab171529) to all wells. The OD of the ELISA plates were read at 450 nm on an ELx808 BioTek plate reader (BioTek, Winooski, VT, USA). The cut-off for a sample being called positive was determined as follows: Average OD of negative serum + 3X standard deviation. Everything above this cut-off was considered positive.

### Gross pathology and histopathology

During *postmortem* examination, the head including the entire upper respiratory tract and central nervous system (brain), trachea and lower respiratory tract, lymphatic and cardiovascular systems, GI tract and urogenital system, and integument were evaluated. CSF was collected with a syringe and needle via the atlanto-occipital (C0-C1) joint. Lungs were evaluated for gross pathology such as edema, congestion, discolouration, atelectasis, and consolidation. Tissue samples from the respiratory tract, nasal turbinates (rostral and deep), trachea (multiple levels; Figure S1B) and all 6 lung lobes (Figure S1C), GI (stomach, small and large intestine) and various other organs and tissues (spleen, kidney, liver, heart, tonsils, tracheo-bronchial and mesenteric lymph nodes, brain including olfactory bulb, and bone marrow) were collected and either fixed in 10% neutral-buffered formalin for histopathologic examination or frozen for RT-qPCR testing. Tissues were fixed in formalin for 7 days, then were transferred to 70% ethanol (ThermoFisher) prior to trimming for embedding. Tissues were routinely processed and stained with hematoxylin and eosin following standard procedures within the histology laboratories of the Kansas State Veterinary Diagnostic Laboratory (KSVDL) and the Louisiana Animal Disease Diagnostic Laboratory (LADDL). Several veterinary pathologists independently examined slides and were blinded to the treatment groups.

### SARS-CoV-2-specific RNAscope^®^ in situ hybridization (RNAscope^®^ ISH)

For RNAscope^®^ ISH, an anti-sense probe targeting the spike (S; nucleotide sequence: 21,563-25,384) of SARS-CoV-2, USA-WA1/2020 isolate was designed (Advanced Cell Diagnostics [ACD], Newark, CA, USA) and used as previously described [23]. Four-micron sections of formalin-fixed paraffin-embedded tissues were mounted on positively charged Superfrost^®^ Plus Slides (VWR, Radnor, PA, USA). The RNAscope^®^ ISH assay was performed using the RNAscope 2.5 HD Red Detection Kit (ACD) as previously described [23,24]. Briefly, deparaffinized sections were incubated with a ready-to-use hydrogen peroxide solution for 10 min at RT and subsequently subjected to Target Retrieval for 15 min at 98–102°C in 1X Target Retrieval Solution. Tissue sections were dehydrated in 100% ethanol for 10 min and treated with Protease Plus for 20 min at 40°C in a HybEZ^™^ oven (ACD). Slides were subsequently incubated with a ready-to-use probe mixture for 2 h at 40°C in the HybEZ^™^ oven, and the signal amplified using a specific set of amplifiers (AMP 1-6 as recommended by the manufacturer). The signal was detected using a Fast-Red solution (Red B: Red A in a 1:60 ratio) for 10 min at RT (RT). Slides were counterstained with 50% Gill hematoxylin I (Sigma Aldrich) for 2 min, and bluing performed with a 0.02% ammonium hydroxide in water. Slides were finally mounted with Ecomount^®^ (Biocare, Concord, CA, USA).

### SARS-CoV-2-specific immunohistochemistry (IHC)

For IHC, four-micron sections of formalin-fixed paraffin-embedded tissue were mounted on positively charged Superfrost^®^ Plus slides and subjected to IHC using a SARS-CoV-2-specific anti-nucleocapsid mouse monoclonal antibody (clone 6F10, BioVision, Inc., Milpitas, CA, USA) as previously described [23]. Briefly, IHC was performed using the automated BOND-MAX and the Polymer Refine Red Detection kit (Leica Biosystems, Buffalo Grove, IL, USA), as previously described [24]. Following automated deparaffinization, heat-induced epitope retrieval (HIER) was performed using a ready-to-use citrate-based solution (pH 6.0; Leica Biosystems) at 100°C for 20 min. Sections were then incubated with the primary antibody (diluted at 1 μg/ml in Antibody Diluent [Dako, Carpinteria, CA]) for 30 min at RT, followed by a polymer-labeled goat anti-mouse IgG coupled with alkaline phosphatase (30 min; Powervision, Leica Biosystems). Fast Red was used as the chromogen (15 min), and counterstaining was performed with hematoxylin. Slides were mounted with a permanent mounting medium (Micromount^®^, Leica Biosystems).

## Results

### SARS-CoV-2-infected cats remain subclinical

Body temperature and clinical signs were recorded daily. No remarkable clinical signs were observed over the course of the study. Body temperatures of principal and sentinel cats remained mostly within normal range, except for the principal infected cats at 2 DPC [Figure S2A]. Body weights of all cats increased throughout the study as expected for young animals without clinical disease [Figure S2B]. Complete blood counts and serum biochemistry were performed on days −1, 1, 3, 5, 7, 10, 14, and 21 DPC for the principal infected cats, and 0, 2, 4, 6, 9, 13 and 20 days post co-mingling (DPCo) for the sentinels. Overall, no significant changes in most blood cell parameters or serum biochemistry were observed. White blood cell (WBC) counts remained within normal limits for most animals during the course of the study. No significant changes were observed in serum biochemical analytes except elevated alkaline phosphatase (ALP) levels in many animals starting at 5 DPC in the sentinels and after 7 DPC in the principal infected animals [Figure S2C], which might indicate growth of subadult animals.

### SARS-CoV-2 RNA found throughout the respiratory tract

**Figure 2.**
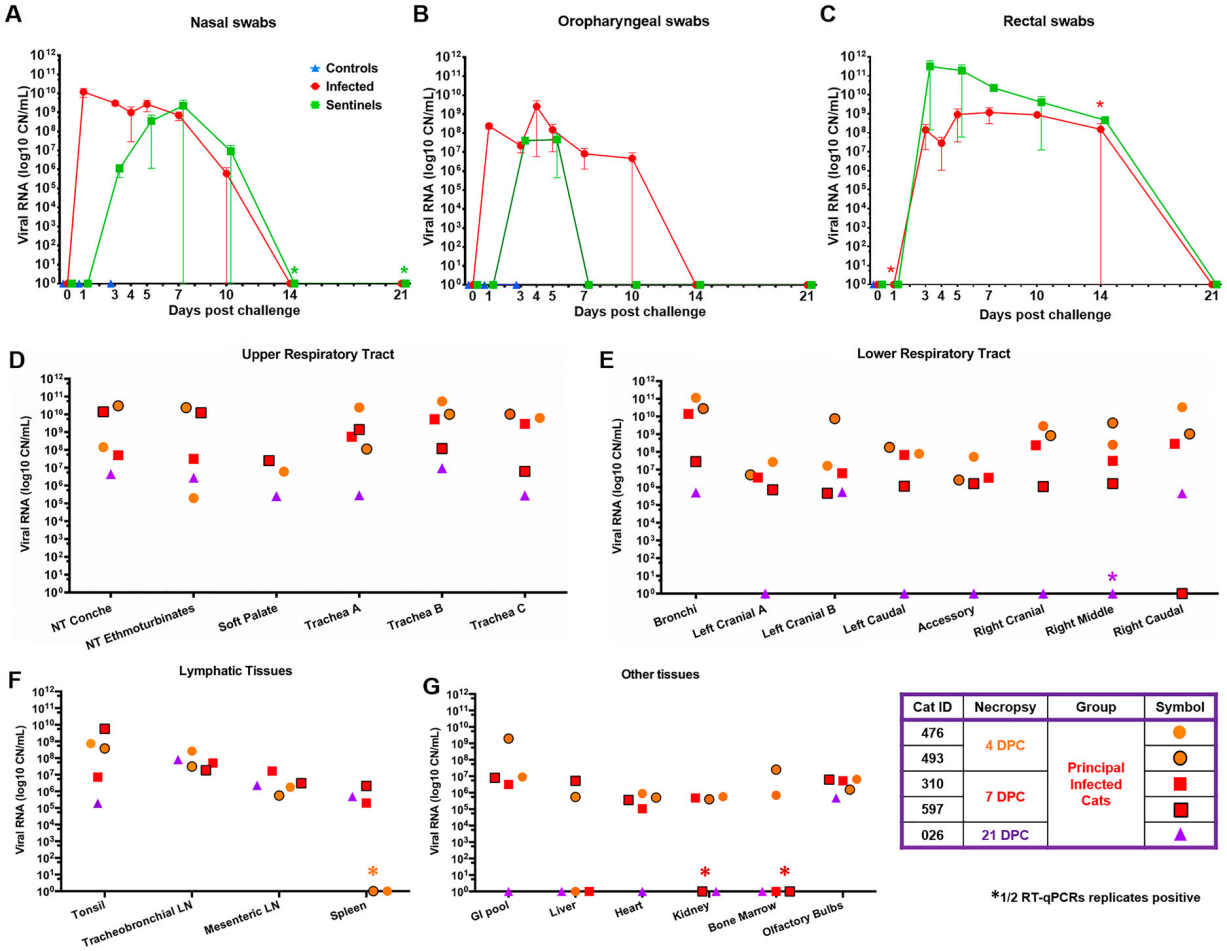
Shedding and presence of SARS-CoV-2 RNA in tissues. RT-qPCR was performed on nasal (A), oropharyngeal (B) and rectal swabs (C) collected from principal infected and sentinel cats over the course of the 21-day study, as well as, on the upper respiratory tract (D), lower respiratory tract (E), lymphatic (F) and other various tissues (G) from principal infected cats necropsied at 4, 7 and 21 days post challenge (DPC). Average viral copy number (CN) per mL or per mg tissues are shown. Astrisks (*) indicate 1 out of 2 of the RT-qPCR reactions were below the limit of detection. LN = lymph node; GI = gastrointestinal.

SARS-CoV-2 RNA was detected in nasal swabs of the principal infected cats at 1 through 10 DPC (Figure 2(A)). The nasal swabs of contact animals became RNA positive for SARS-CoV-2 starting at day 2 DPCo (i.e. 3 DPC) and remained positive up to 9 DPCo (Figure 2(A)). The oropharyngeal swabs were RNA positive starting at 1 DPC through 10 DPC for the principals and 2 DPCo through 4 DPCo for the sentinels (Figure 2(B)).

Viral RNA was also detected in respiratory tract tissues in principal infected animals (Figure 2(D,E)). Fresh tissues collected during *postmortem* examination from the nasal cavity, trachea, bronchi and all lung lobes were RNA positive for all animals at 4 and 7 DPC (Figure 2(D,E)). Viral RNA levels in the lungs tended to be lower than the upper respiratory tract for cats necropsied at 7 DPC (Figure 2(E)). At 21 DPC viral RNA was detected within the upper respiratory samples, as well as bronchi and the right caudal and left cranial lung lobes (Figure 2(D,E)). Nasal washes and BALF collected at necropsy from all principal infected cats examined at 4 and 7 DPC were RNA positive, but negative from the cat evaluated at 21 DPC (Table 1).

**Table 1.** Viral RNA (copy number/mL) detected in nasal washes, bronchoalveolar lung fluid (BALF), cerebrospinal fluid (CSF) and urine.

Cat ID#	DPC	Nasal washes	BALF	CSF	Urine
476	4	6.5E+08	5.9E+08	2.5E+04*	ND
493	4	7.5E+08	3.2E+08	ND	ND
310	7	6.3E+07	2.0E+08	3.6E+06	ND
597	7	5.6E+07	4.6E+05	ND	ND
026	21	ND	ND	ND	ND

ND = not determined; *1/2 RT-qPCR reactions below limit of detection.

**Figure 3.**
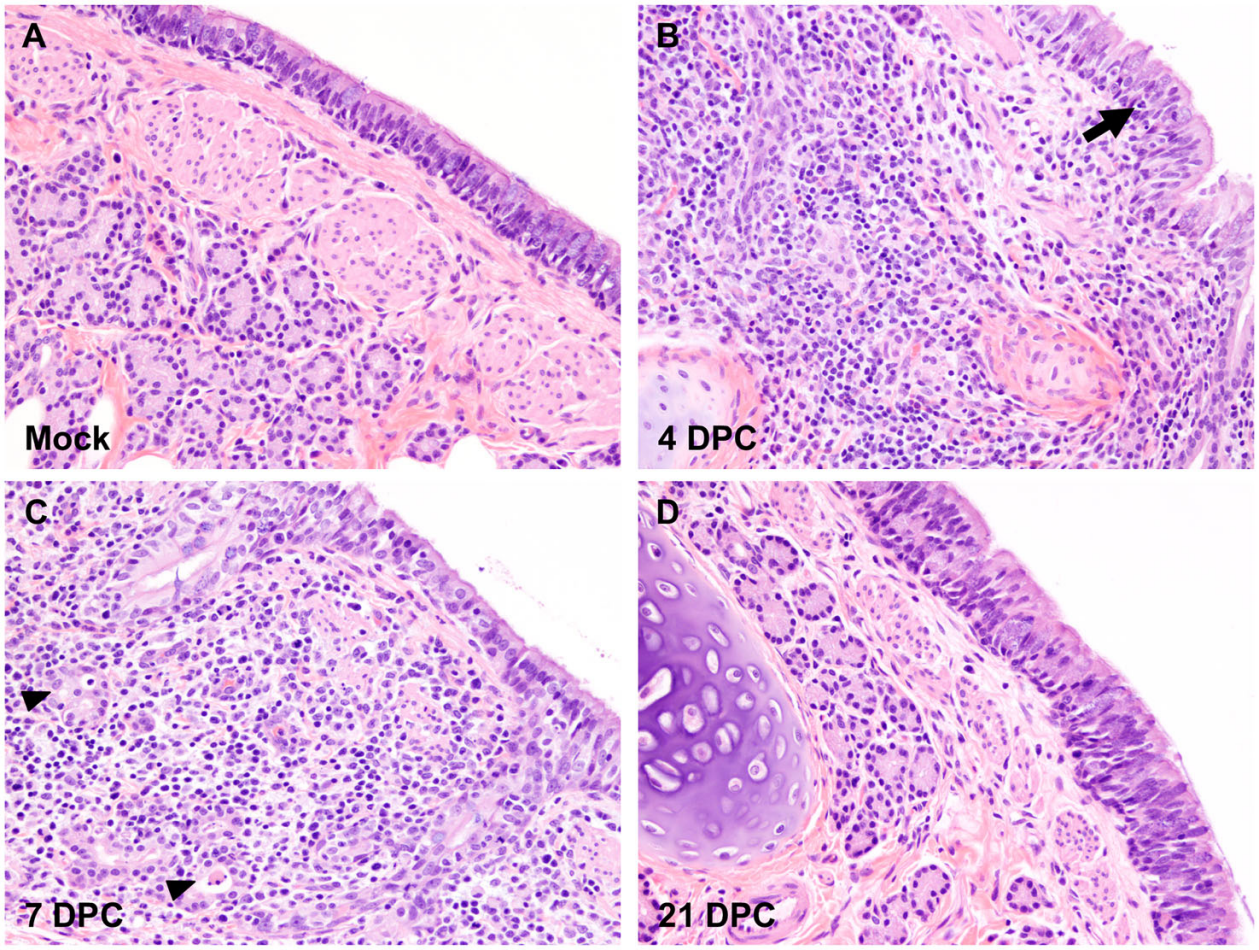
Histopathology of bronchi. Histological findings in the main bronchi of mock (A) and SARS-CoV-2 experimentally infected (B-D) cats. Histologic changes and their progression are similar to those observed in the trachea, with multifocal, widespread, mild to moderate lymphocytic and neutrophilic adenitis noted at 4 DPC (B) and 7 DPC (C). Necrotic debris within distorted submucosal glands are indicated with arrowheads (C), and few transmigrating lymphocytes are indicated with an arrow (B). No histologic changes are noted at 21 DPC (D). H&E. Total magnification: 200X.

Gross pathology of the respiratory tract was assessed during *postmortem* examination for each animal at 4, 7 and 21 DPC and demonstrated various degrees and distribution of edema, discolouration, congestion and atelectasis (data not shown). Histologically, the pathological changes were limited to the upper and lower airways (larynx, trachea, and main, lobar and segmental bronchi of the lungs) of SARS-CoV-2 principal infected cats. Pathological findings are characterized by multifocal lymphocytic and neutrophilic tracheobronchoadenitis of seromucous glands of the lamina propria and submucosa of the trachea and bronchi. Changes range from minimal to mild at 4 DPC and progress to mild to moderate by 7 DPC (Figure 3, Figure S3). Affected submucosal glands and associated ducts were variably distended, lined by attenuated epithelium, and contain necrotic cell debris. More severely affected glands are poorly delineated, and disrupted by mild to moderate numbers of infiltrating lymphocytes, macrophages and plasma cells, and few neutrophils (Figure 3, Figure S3). No significant pathology was identified elsewhere in the pulmonary parenchyma of SARS-CoV-2-infected cats on 4 and 7 DPC. No significant histologic changes were noted in the respiratory tract at 21 DPC, with the submucosal architecture of the trachea and bronchi being unremarkable and within normal limits (Figure 3, Figure S3).

**Figure 4.**
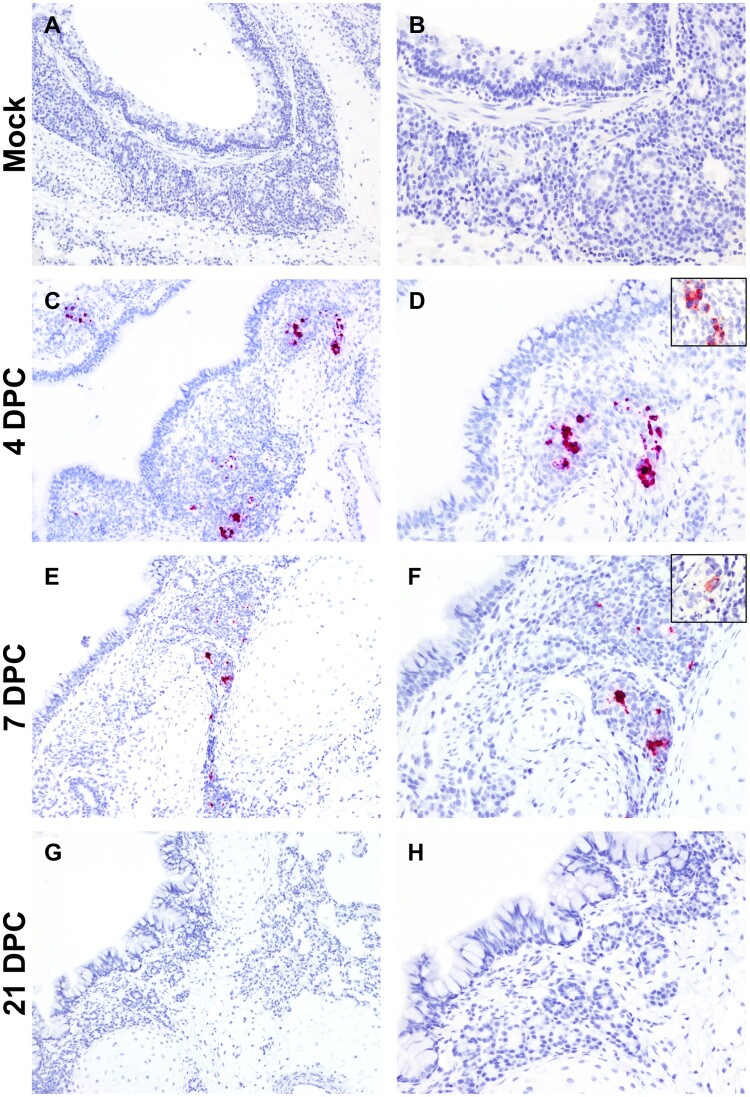
SARS-CoV-2 RNA and antigen detection in bronchi. SARS-CoV-2 tropism in bronchi of mock (A and B) experimentally (C-H) infected cats determined by S-specific RNAscope^®^ *in situ* hybridization (Fast Red) and anti-N-specific immunohistochemistry (IHC; Fast Red). The viral tropism is limited to glandular and ductular epithelial cells of multifocal, scattered submucosal glands. Viral RNA is detected within infected cells at 4 days post-challenge (DPC; C and D) and, to a lower degree at 7 DPC (E and F). Few scattered glandular epithelial cells are positive for SARS-CoV-2 N antigen by IHC (D and F, insets). No viral RNA or antigen is detected at 21 DPC (G and H). Total magnification: 100X (A, C, E and G) and 200X (B, D, F, H).

The cellular tropism, distribution and abundance of SARS-CoV-2 were also investigated via the detection of viral RNA and viral antigen by RNAscope^®^ ISH and IHC, respectively. Presence of viral RNA and antigen correlated with the histological changes observed in the airways and were detected within epithelial cells of submucosal glands and associated ducts at 4 and 7 DPC (Figure 4, Figure S4). SARS-CoV-2-positive submucosal glands were more frequently observed at 4 DPC compared to 7 DPC but not at 21 DPC (Figure 4, Figure S4). No viral RNA or antigen were detected within lining epithelial cells or elsewhere in the pulmonary parenchyma, including smaller airways and alveoli, at 4, 7 or 21 DPC.

### SARS-CoV-2 RNA found throughout non-respiratory organs and tissues

SARS-CoV-2 RNA was detected in rectal swabs starting at 3 DPC for principal infected cats and 2 DPCo for sentinel cats and were found positive up to 14 DPC/13 DPCo (Figure 2(C)). Urine collected directly from the bladder during *postmortem* examination of cats sacrificed at 4, 7, 21 DPC was negative by RT-qPCR (Table 1). Viral RNA was detected in the GI tract and other organs and tissues in principal infected animals (Figure 2(F,G)). Tonsils, lymph nodes and olfactory bulbs of all cats were positive on 4, 7, and 21 DPC with the highest RNA levels detected in the tonsils and lymph nodes. Spleen was negative on 4 DPC, but positive on 7 and 21 DPC. RNA was present in the pooled tissue from the GI tract and heart in all animals on 4 and 7 DPC. Liver, heart, kidney, and bone marrow were occasionally positive on different DPC. CSF was positive from 1 of the 2 cats necropsied at 4 DPC and 1 of 2 cats necropsied at 7 DPC, but not at 21 DPC (Figure 2(G), and Table 1). Blood from all cats collected at −1, 1, 3, 5, 7, 10, 14 and 21 DPC was negative for SARS-CoV-2 RNA. Gross and histopathological evaluation of the GI tract, the cardiovascular and central nervous system as well as major visceral organs and lymphoid tissues revealed no pathological changes at any DPC.

### Seroconversion of cats after SARS-CoV-2 infection

Sera at different DPC was tested for the presence of SARS-CoV-2 specific antibodies. Virus neutralizing antibodies were detected in sera from all principal infected and sentinel cats at 7 (not sentinel), 10, 14 and 21 DPC, with neutralizing titres ranging from 1:20 to 1:320 (Table 2). Antibodies against the N protein were detected in principal cats starting at 5 DPC and in the two sentinels at 13 DPCo (Table 2). Similarly, antibodies against RBD protein were detected in principal cats at starting at 5 DPC and in the sentinels starting at 13 DPCo.

**Table 2.** Felines develop SARS-CoV-2 specific and virus neutralizing antibodies.

	Anti-N antibodies					Anti-RBD antibodies					Virus neutralizing antibodies				
DPC	5	7	10	14	21	5	7	10	14	21	5	7	10	14	21
*Principals*	* *	* *	* *	* *	* *	* *	* *	* *	* *	* *	* *	* *	* *	* *	* *
310	−	−	NA	NA	NA	−	−	NA	NA	NA	<1:20	1:80	NA	NA	NA
597	−	−	NA	NA	NA	−	−	NA	NA	NA	<1:20	1:160	NA	NA	NA
026	+	+	+	+	+	−	+	+	+	+	<1:20	1:80	1:320	1:80	1:40
328	−	+	+	+	NA	+	+	+	+	NA	<1:20	1:20	1:160	1:160	1:160
*Sentinels*	* *	* *	* *	* *	* *	* *	* *	* *	* *	* *	* *	* *	* *	* *	* *
272	−	−	−	+	+	−	−	−	−	+	<1:20	<1:20	1:160	1:160	1:160
903	−	+/−	+/−	+	+	−	−	−	+	+	<1:20	<1:20	1:80	1:160	1:160

NA = not available; N = nucleocapsid; RBD = receptor binding domain of spike protein; +/− = OD close to cut-off.

## Discussion

In this study, we explored in-depth the infection, associated disease and transmission dynamics in 4–5-months old domestic cats. While the minimum infectious dose of SARS-CoV-2 for humans, cats or other susceptible animals is not currently known, in our study we administered a relatively high infectious dose via multiple natural infection routes, to achieve sufficient virus exposure for primary infection. Compared to the SARS-CoV-2 study in cats by Shi and colleagues (2020), we used a 1 log higher infectious dose administered orally and intranasally simultaneously. The detection of high levels of viral RNA from swab samples and in various organs and tissues, along with mild to moderate histologic changes in trachea and bronchi associated with viral RNA and antigen, and the development of SARS-CoV-2-specific antibodies demonstrates that cats were productively infected, without developing any obvious clinical signs. Furthermore, the principal infected cats were able to transmit the virus to sentinel animals within 2 days of contact housing similar to previous reports [20,25]. Shedding of virus through the respiratory and GI tract are most likely responsible for the transmission to the sentinel animals. Shi and coworkers (2020) determined that airborne transmission of SARS-CoV-2 among cats is possible but not highly effective.

Our and previous results [20,25], as well as reports of cats in households with COVID-19 patients [18,19] show that felines are susceptible to SARS-CoV-2 infection and could be potential virus reservoirs. Consistent with our results, previous studies [20,25] also reported no obvious clinical signs in SARS-CoV-2 infected cats which were older than 4 months. We detected high viral RNA levels in many tissues tested at 4 and 7 DPC, with reduced levels (upper respiratory tract, lymphoid tissues, CNS) or clearing by 21 DPC. Since no virus was detected in blood, it remains to be studied how the virus reaches and infects non-respiratory tissues including the CNS. Shi and colleagues [20] detected viral RNA and infectious virus throughout upper and lower respiratory tracts in juvenile (70–100 days old) and subadult cats (6–9 months old) at 3 DPC, but it was cleared from most lung tissues of subadult cats by 6 DPC. However, in juvenile cats, virus was still present at 6 DPC in the lower respiratory tract [20]. In contrast, no virus was detected in other organs of any of these cats which included brain, heart, submaxillary lymph nodes, kidney, spleen, liver, and pancreas at 3 or 6 DPC [20]. Similar to Shi and colleagues (2020) who detected virus in the small intestine of most of the animals, we found shedding of viral RNA in rectal swabs up to 14 DPC and in pooled GI tract tissues on 4 and 7 DPC. In contrast, Halfmann and coworkers (2020) found all rectal swabs from 4-5 month old animals to be virus negative. These differences may be explained by the age of the cats and and/or different virus strains used. SARS-CoV-2 associated pathological changes in juvenile and subadult infected cats were reported, but more severe pathology was found in the juvenile cats [20].

Similarly, our results show that subadult cats had mild to moderate histologic alterations identified as tracheobronchoadenitis within the airways. The macroscopic lung lesions observed were most likely due to the euthanasia with barbiturates. Importantly, all SARS-CoV-2 infected cats (principals and sentinels) in our study mounted an antiviral and neutralizing antibody response during the 21-day observation period. The development of an early serological response starting at 5–7 DPC in some of the principal infected cats could be indicative of the high infectious dose, compared to sentinels which developed antibodies around 10–14 DPC. Other studies reported detection of virus-specific and/or neutralizing antibodies also in all infected animals [20,25]. None of these studies detected virus or viral RNA in the blood.

Experimental infection of cats with SARS-CoV [9,10] and SARS-CoV-2 [20] reveal histological changes within the airways after various DPC. Similarly, our SARS-CoV-2 infection of subadult cats demonstrated mild to moderate neutrophilic and lymphocytic tracheobronchoadenitis with associated intralesional detection of viral RNA and viral antigen. While SARS-CoV antigen was identified in cat tracheal and bronchial epithelial cells [9,10], we found that the epithelial cells of trachea and bronchi seem non-permissive to SARS-CoV-2 replication in subadult cats as demonstrated by the lack of viral RNA and antigen; this correlated with the absence of histologic alterations on the surface epithelium. These findings are in partial disagreement to those of another recent cat study [20], where mild histologic alterations in the tracheal lumen and epithelium were reported in the absence of detectable viral antigen. Interestingly, SARS-CoV-2 infected subadult cats did not present histological changes within the small airways or the pulmonary parenchyma consistent with interstitial pneumonia or diffuse alveolar damage, such as inflammatory infiltrates within alveoli, intra-alveolar fibrin or hyaline membranes, or pneumocyte type II hyperplasia. Additionally, there is no evidence of SARS-CoV-2 infection within pneumocytes or alveolar macrophages as demonstrated by the absence of viral RNA and antigen. These findings correlate with the absence of clinically evident respiratory disease following experimental SARS-CoV-2 infection. While a recent study evaluating the susceptibility of juvenile cats to SARS-CoV-2 [20] suggested the occurrence of histologic alterations in the pulmonary vasculature and alveolar spaces, none of these changes were noted in our study. Additional investigations are needed to determine whether these differences are due to the breed and age of the cats, the virus isolate used or other factors.

Together these findings warrant COVID-19 screening of felines for epidemiological purposes and for implementation of mitigation strategies; they also point towards nasal swabs/washes and rectal swabs as appropriate diagnostic samples. This information will be important for providing appropriate veterinary care for infected cats and cats in their surroundings, for protection of veterinary personnel, animal caretakers and pet owners, and for applying quarantine measures to prevent transmission between felines, people and potentially other susceptible animals. The ease of transmission between domestic cats indicates a significant public health necessity to investigate a potential human-cat-human transmission chain. It is also critical that pet owners are educated on the risks and preventative measures in order to calm fears and discourage animal abandonment.

Although asymptomatic, cats can be productively infected and readily transmit SARS-CoV-2 to other susceptible cats, and thus may serve as potential models for asymptomatic COVID-19 infections in humans. Cats could offer a model for testing vaccines and antiviral candidates for companion animals and for drugs with a problematic pharmacokinetic profile in rodents, ferrets or nonhuman primates. However, a preclinical animal model that mimics the clinical symptoms and disease observed in severe COVID-19 patients is still needed to improve the evaluation of vaccines, antiviral drugs and other therapies.

Further research is needed to adapt models to recapitulate severe disease observed in humans. One area to explore is the effect of age on clinical outcome. Only cats less than 1-year-old were evaluated in this and previous studies [20,25]; however, SARS-CoV-2 infection in adult and old cats, and questions regarding re-infection of cats were not explored in these studies. Recent non peer-reviewed work by Bosco-Lauth and colleagues (2020) investigated experimental SARS-CoV-2 challenge and transmission in adult cats 5–8 years old, and re-infection after 28 DPC [26]. That study demonstrated that adult cats become infected without clinical signs and with pathology limited to respiratory airways, can readily transmit the virus to naïve cats, and appear to be protected from reinfection [26]. Studies to better understand the mechanisms of infection and the range of symptoms and pathology associated with SARS-CoV-2 in various preclinical models of COVID-19 are critical for the development of vaccines and treatments for this disease.

## Supplementary Material

Supplemental Material

## References

[1] Fehr AR, Perlman S. Coronaviruses: an overview of their replication and pathogenesis. Methods Mol Biol. 2015;1282:1–23. 25720466 10.1007/978-1-4939-2438-7_1 PMC4369385

[2] Gorbalenya AE, Baker SC, Baric RS, et al. The species severe acute respiratory syndrome-related coronavirus: classifying 2019-nCoV and naming it SARS-CoV-2. Nature Microbiology. 2020;5(4):536–544. 10.1038/s41564-020-0695-z PMC7095448 32123347

[3] Fung TS, Liu DX. Human Coronavirus: host-pathogen Interaction. Annu Rev Microbiol. 2019;73:529–557. 31226023 10.1146/annurev-micro-020518-115759

[4] Saif LJ. Animal coronaviruses: what can they teach us about the severe acute respiratory syndrome? Rev Sci Tech. 2004;23(2):643–660. 15702725 10.20506/rst.23.2.1513

[5] Woo PC, Lau SK, Li KS, et al. Genetic relatedness of the novel human group C betacoronavirus to Tylonycteris bat coronavirus HKU4 and Pipistrellus bat coronavirus HKU5. Emerg Microbes Infect. 2012;1(11):e35. 26038405 10.1038/emi.2012.45 PMC3630921

[6] Drexler JF, Corman VM, Drosten C. Ecology, evolution and classification of bat coronaviruses in the aftermath of SARS. Antiviral Res. 2014;101:45–56. 24184128 10.1016/j.antiviral.2013.10.013 PMC7113851

[7] Corman VM, Muth D, Niemeyer D, et al. Hosts and Sources of Endemic human coronaviruses. Adv Virus Res. 2018;100:163–188. 29551135 10.1016/bs.aivir.2018.01.001 PMC7112090

[8] de Wit E, van Doremalen N, Falzarano D, et al. SARS and MERS: recent insights into emerging coronaviruses. Nat Rev Microbiol. 2016;14(8):523–534. 27344959 10.1038/nrmicro.2016.81 PMC7097822

[9] Martina BE, Haagmans BL, Kuiken T, et al. Virology: SARS virus infection of cats and ferrets. Nature. 2003;425(6961):915. 14586458 10.1038/425915a PMC7094990

[10] van den Brand JM, Haagmans BL, Leijten L, et al. Pathology of experimental SARS coronavirus infection in cats and ferrets. Vet Pathol. 2008;45(4):551–562. 18587105 10.1354/vp.45-4-551

[11] Zhou P, Yang XL, Wang XG, et al. A pneumonia outbreak associated with a new coronavirus of probable bat origin. Nature. 2020;579(7798):270–273. 32015507 10.1038/s41586-020-2012-7 PMC7095418

[12] Li Q, Guan X, Wu P, et al. Early transmission dynamics in Wuhan, China, of Novel Coronavirus-infected pneumonia. N Engl J Med. 2020;382(13):1199–1207. 31995857 10.1056/NEJMoa2001316 PMC7121484

[13] Andersen KG, Rambaut A, Lipkin WI, et al. The proximal origin of SARS-CoV-2. Nat Med. 2020;26(4):450–452. 32284615 10.1038/s41591-020-0820-9 PMC7095063

[14] Zhang T, Wu Q, Zhang Z. Pangolin homology associated with 2019-nCoV. bioRxiv. 2020:2020.02.19.950253.

[15] Lakdawala SS, Menachery VD. The search for a COVID-19 animal model. Science. 2020;368(6494):942–943. 32467379 10.1126/science.abc6141

[16] Hernández M, Abad D, Eiros JM, et al. Are animals a Neglected transmission Route of SARS-CoV-2? Pathogens. 2020;9(6):480. doi:10.3390/pathogens9060480. PMID: 32570713; PMCID: PMC7350367. 32570713 PMC7350367

[17] Oreshkova N, Molenaar R-J, Vreman S, et al. SARS-CoV2 infection in farmed mink, Netherlands, April 2020. bioRxiv. 2020:2020.05.18.101493. 10.2807/1560-7917.ES.2020.25.23.2001005 PMC7403642 32553059

[18] Newman A, Smith D, Ghai RR, et al. First reported Cases of SARS-CoV-2 infection in companion animals - New York, March-April 2020. MMWR Morb Mortal Wkly Rep. 2020;69(23):710–713. 32525853 10.15585/mmwr.mm6923e3 PMC7315787

[19] Leroy EM, Ar Gouilh M, Brugère-Picoux J. The risk of SARS-CoV-2 transmission to pets and other wild and domestic animals strongly mandates a one-health strategy to control the COVID-19 pandemic. One health (Amsterdam, Netherlands). 2020:100133. 10.1016/j.onehlt.2020.100133 PMC7194722 32363229

[20] Shi J, Wen Z, Zhong G, et al. Susceptibility of ferrets, cats, dogs, and other domesticated animals to SARS-coronavirus 2. Science. 2020;368(6494):1016–1020. 32269068 10.1126/science.abb7015 PMC7164390

[21] Cleary SJ, Pitchford SC, Amison RT, et al. Animal models of mechanisms of SARS-CoV-2 infection and COVID-19 pathology. Br J Pharmacol. 2020. doi:10.1111/bph.15143. Epub ahead of print. PMID: 32462701; PMCID: PMC7283621. PMC7283621 32462701

[22] Hierholzer JC, Killington RA. 2 - Virus isolation and quantitation A2 - Mahy, Brian WJ. In: Kangro HO, editor. Virology Methods Manual. London: Academic Press; 1996. p. 25–46.

[23] Carossino M, Ip HS, Richt JA, et al. Detection of SARS-CoV-2 by RNAscope® in situ hybridization and immunohistochemistry techniques. Arch Virol. 2020;165(10):2373–2377. doi:10.1007/s00705-020-04737-w 32761270 PMC7406679

[24] Carossino M, Dini P, Kalbfleisch TS, et al. Equine arteritis virus long-term persistence is orchestrated by CD8+ T lymphocyte transcription factors, inhibitory receptors, and the CXCL16/CXCR6 axis. PLoS Pathog. 2019;15(7):1–42, e1007950. 10.1371/journal.ppat.1007950 PMC6692045 31356622

[25] Halfmann PJ, Hatta M, Chiba S, et al. Transmission of SARS-CoV-2 in domestic cats. N Engl J Med. 2020;383(6):592–594. 32402157 10.1056/NEJMc2013400 PMC9678187

[26] Bosco-Lauth AM, Hartwig AE, Porter SM, et al. Pathogenesis, transmission and response to re-exposure of SARS-CoV-2 in domestic cats. bioRxiv. 2020:2020.05.28.120998.

