# Supplementary figures and images for: SARS-CoV-2 infection, disease and transmission in domestic cats
Source: Emerg Microbes Infect. 2020 Oct 25;9(1):2322–32. doi: 10.1080/22221751.2020.1833687 (PMC7594869; doi:10.1080/22221751.2020.1833687)

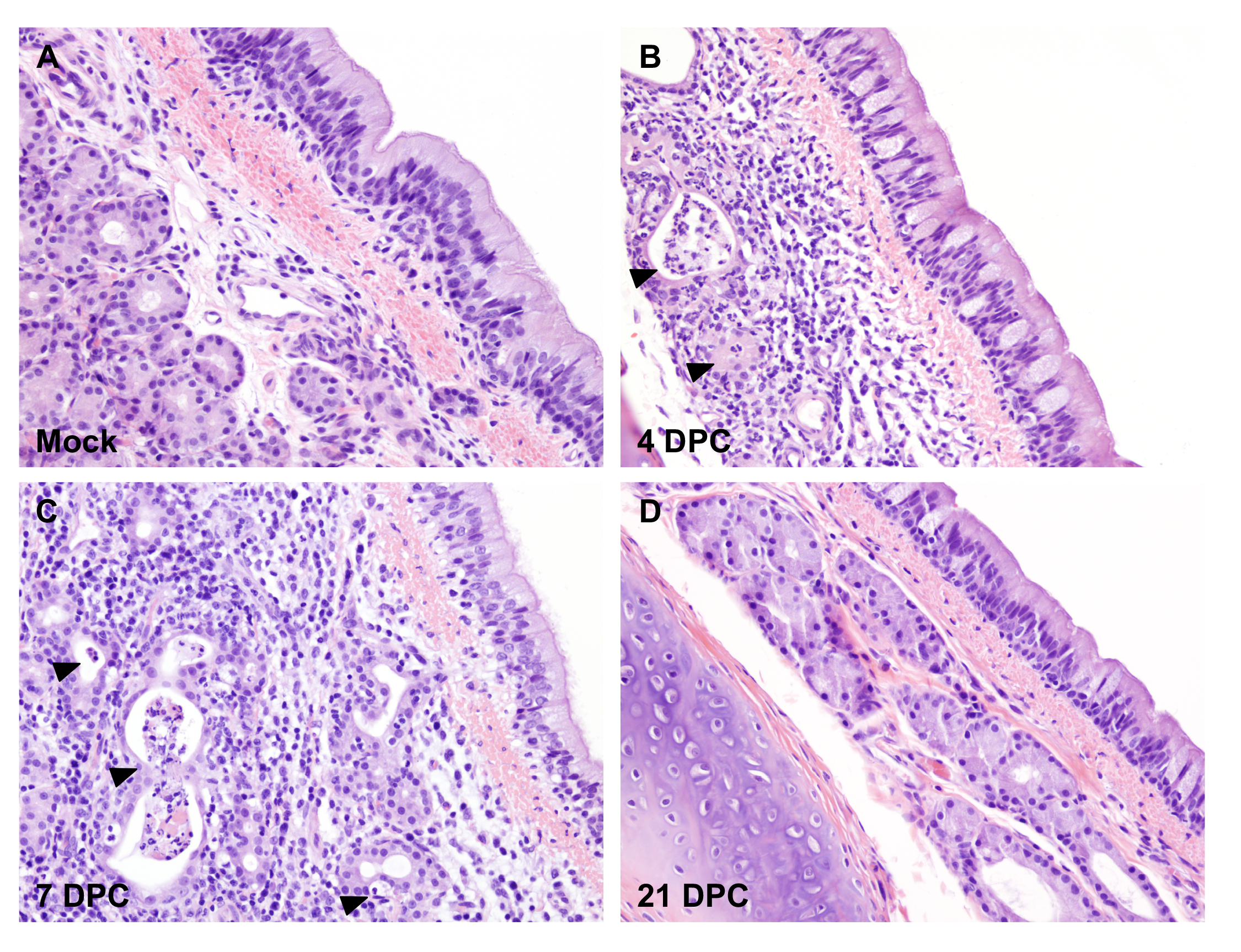

Supplement: Supplemental Material [file TEMI_A_1833687_SM7602.zip › TEMI 1833687_Supplementary figs/Figure S3_HE_panel1_trachea.tif]

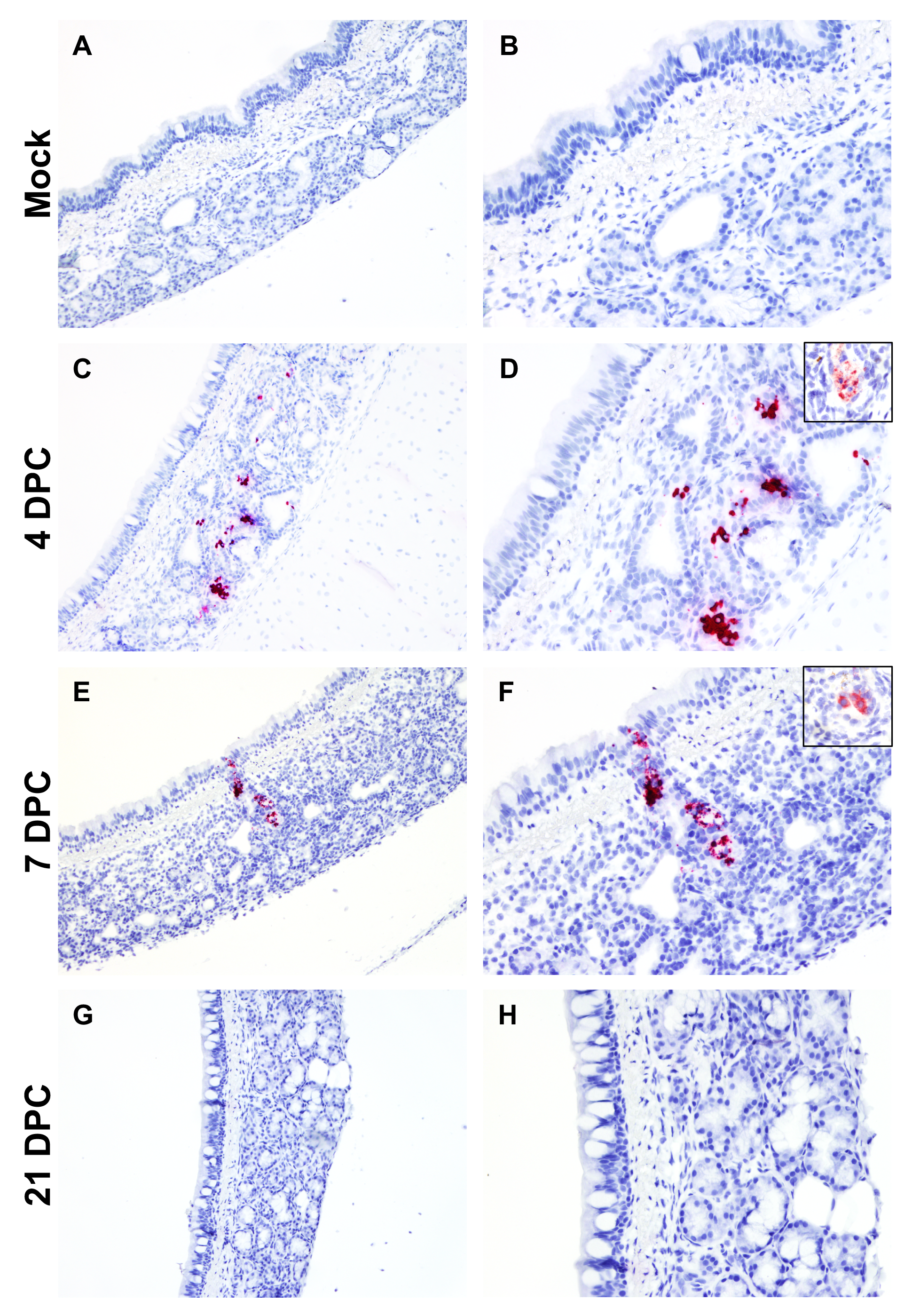

Supplement: Supplemental Material [file TEMI_A_1833687_SM7602.zip › TEMI 1833687_Supplementary figs/Figure S4_ISHandIHC_panel1_trachea.tif]

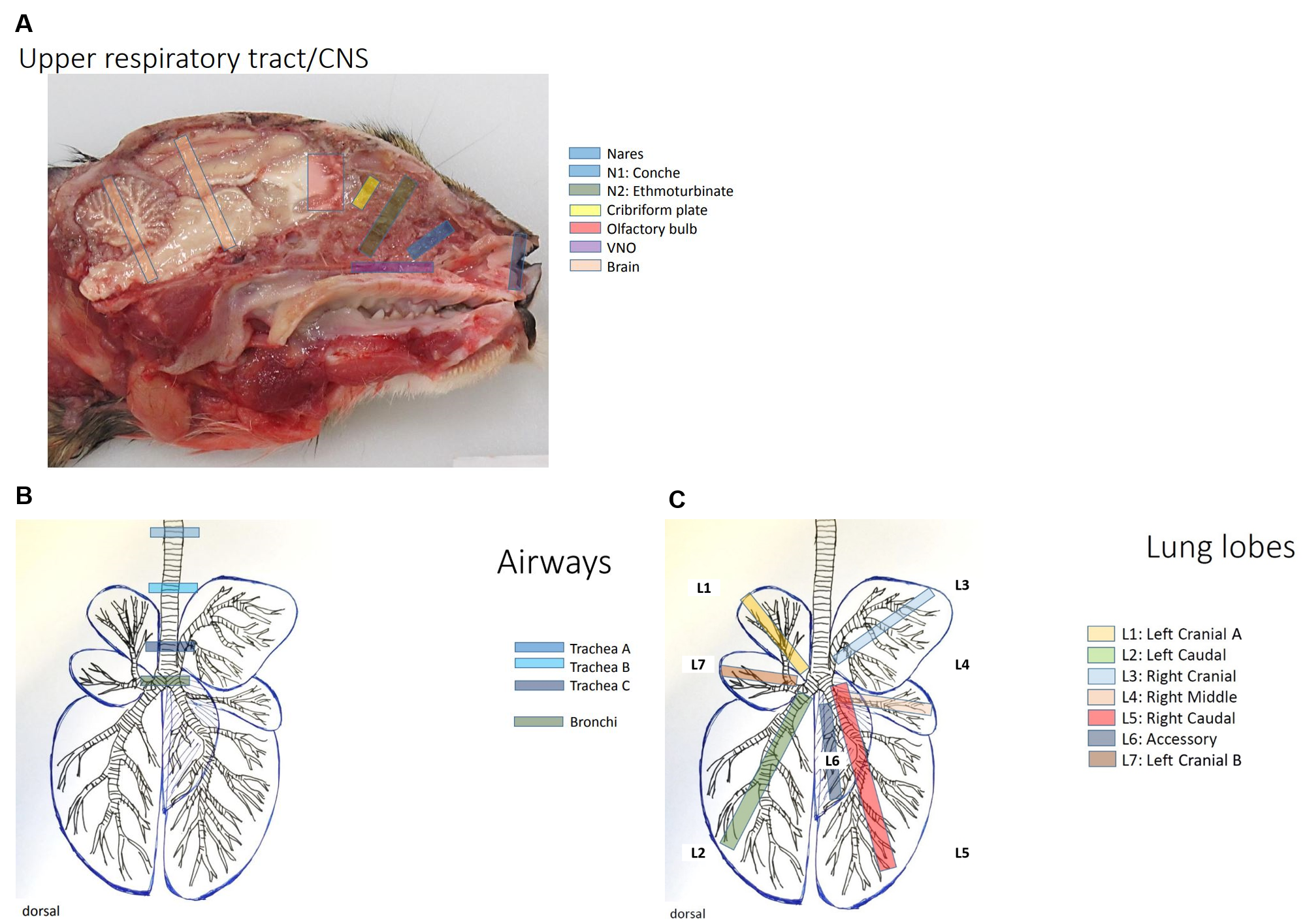

Supplement: Supplemental Material [file TEMI_A_1833687_SM7602.zip › TEMI 1833687_Supplementary figs/S1-proof edit.tiff]
